# Novel TRKB agonists activate TRKB and downstream ERK and AKT signaling to protect Aβ-GFP SH-SY5Y cells against Aβ toxicity

**DOI:** 10.18632/aging.204306

**Published:** 2022-09-26

**Authors:** Ya-Jen Chiu, Te-Hsien Lin, Kuo-Hsuan Chang, Wenwei Lin, Hsiu Mei Hsieh-Li, Ming-Tsan Su, Chiung-Mei Chen, Ying-Chieh Sun, Guey-Jen Lee-Chen

**Affiliations:** 1 Department of Life Science, National Taiwan Normal University, Taipei 11677, Taiwan; 2 Department of Neurology, Chang Gung Memorial Hospital, Chang Gung University College of Medicine, Taoyuan 33302, Taiwan; 3 Department of Chemistry, National Taiwan Normal University, Taipei 11677, Taiwan

**Keywords:** Alzheimer’s disease, TRKB agonists, Aβ, neuroprotection, therapeutics

## Abstract

Decreased BDNF and impaired TRKB signaling contribute to neurodegeneration in Alzheimer’s disease (AD). We have shown previously that coumarin derivative LM-031 enhanced CREB/BDNF/BCL2 pathway. In this study we explored if LM-031 analogs LMDS-1 to -4 may act as TRKB agonists to protect SH-SY5Y cells against Aβ toxicity. By docking computation for binding with TRKB using 7,8-DHF as a control, all four LMDS compounds displayed potential of binding to domain d5 of TRKB. In addition, all four LMDS compounds exhibited anti-aggregation and neuroprotective efficacy on SH-SY5Y cells with induced Aβ-GFP expression. Knock-down of TRKB significantly attenuated TRKB downstream signaling and the neurite outgrowth-promoting effects of these LMDS compounds. Among them, LMDS-1 and -2 were further examined for TRKB signaling. Treatment of ERK inhibitor U0126 or PI3K inhibitor wortmannin decreased p-CREB, BDNF and BCL2 in Aβ-GFP cells, implicating the neuroprotective effects are via activating TRKB downstream ERK, PI3K-AKT and CREB signaling. LMDS-1 and -2 are blood–brain barrier permeable as shown by parallel artificial membrane permeability assay. Our results demonstrate how LMDS-1 and -2 are likely to work as TRKB agonists to exert neuroprotection in Aβ cells, which may shed light on the potential application in therapeutics of AD.

## INTRODUCTION

Alzheimer’s disease (AD) is the most common disorder that causes dementia. Amyloid β (Aβ)-containing plaques and neurofibrillary tangles deposited in the brains are the main pathological features [1]. Several mutations in the Aβ precursor protein (APP) and presenilin-1 (PS1) and -2 (PS2) genes have been shown to cause familial AD [2]. Aβ deposition is one of the early events in AD pathology [3]. APP is an integral membrane protein that is proteolyzed to generate a peptide containing 39–43 amino acids. The Aβ peptide composed of 42 amino acids (Aβ42) is the main peptide accumulated in the AD brains [4]. Aβ monomer misfolds into oligomers, pre-fibrillar and insoluble fibrillar aggregates that are toxic to neurons [5, 6]. The accumulated misfolded proteins cause neurotoxicity that leads to apoptosis [7, 8]. Thus, clearance of the abnormal Aβ aggregates has been suggested as a therapeutic method to modify disease progression in AD.

Brain-derived neurotrophic factor (BDNF), a neurotrophic factor, is expressed ubiquitously in whole brain and plays a vital role in promoting neural plasticity, neuronal growth and cell survival [9]. BDNF has a high binding affinity to tropomyosin-related kinase B (TRKB), also known as neurotrophic receptor tyrosine kinase 2, to induce TRKB dimerization and phosphorylation of TRKB, and subsequently to activate downstream cascades including, phosphoinositide 3-kinase (PI3K)-AKT serine/threonine kinase (AKT), extracellular signal-regulated kinase (ERK)-cAMP responsive element binding protein 1 (CREB), and phospholipase C-γ1 (PLC-γ1). The TRKB receptor activation would lead to enhanced memory formation and storage as well as neuroplasticity, neurogenesis, neurite outgrowth and neuronal survival [10, 11]. ERK phosphorylates CREB to promote transcription of genes for neuronal survival, neurite outgrowth and neuroplasticity [12], such as BDNF [13] and BCL2 apoptosis regulator (BCL2) [14]. The post-synaptic ERK signal would also enhance the neuroplasticity related long-term potentiation [11].

Decreased BDNF has been shown in several neurodegenerative diseases [12]. Previous reports have shown that BDNF transcription and protein expression is reduced in hippocampus, cortex and Meynert basal ganglion of AD brains [13, 14]. It has been shown that accumulation of Aβ is associated with loss of BDNF [15] and Aβ inhibits protein kinase A to down-regulate the CREB phosphorylation and expression of BDNF [15, 16]. Oligomeric Aβ also interferes with Ras-ERK and PI3K-AKT pathways to aggravate neurotoxicity [17]. Several preclinical studies using AD mouse models have demonstrated that increased BDNF expression rescues neurotoxicity and improves cognitive function [18–20]. Nevertheless, the poor bioavailability of BDNF, or example the short half-life in plasma and the limited blood–brain barrier (BBB) permeability [21], restricts the application of BDNF.

Developing novel small molecule agonists of TRKB receptor is a potential therapeutic strategy for neurodegenerative diseases including AD [22]. To identify compounds acting as TRKB agonists with a good BBB permeability is crucial for developing therapeutics for AD. Our previous studies have demonstrated that a novel coumarin derivative, LM-031, displayed neuroprotective effects by upregulating CREB/BDNF/BCL2 pathway in our Flp-In Aβ-GFP SH-SY5Y cells [23]. In this study, we searched three online databases for LM-031 analogous compounds using compound similarity search software tools and identified four top-scoring compounds LMDS-1 to -4 as the candidate TRKB agonists. To examine their plausible role as TRKB agonists, docking computation was firstly conducted to investigate binding strength and conformation of LM-031 and the four analogs. We then examined if the four potential small TRKB agonists would exert neuroprotective effects and influence the TRKB downstream pathways in Aβ-GFP folding reporter SH-SY5Y cells. The BBB permeability was also examined by using parallel artificial membrane permeability assay (PAMPA).

## RESULTS

### Test compounds

**Figure 1:**
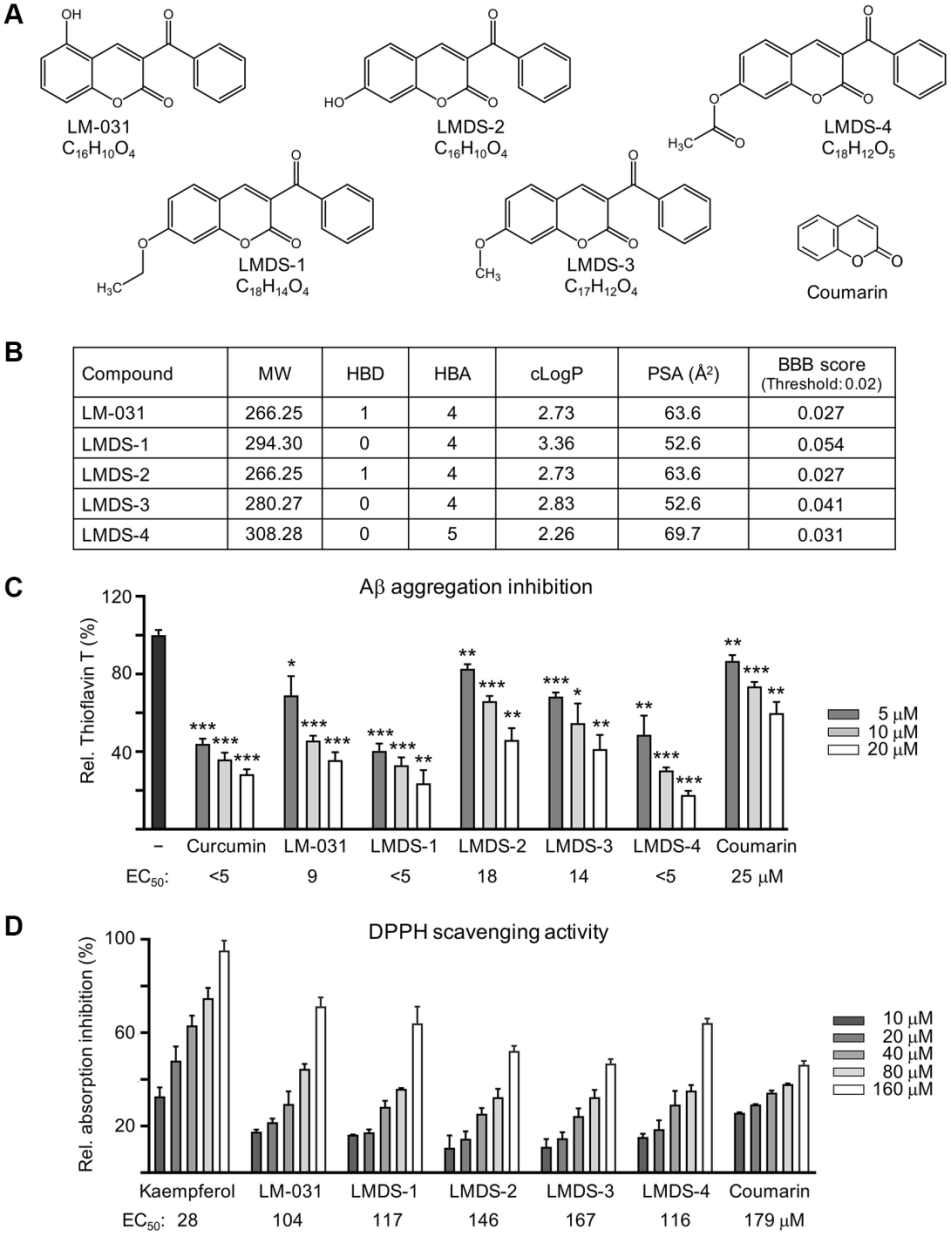
**LM-031 and analogous compounds.** (**A**) Structure and formula of LM-031 and analogs LMDS-1 to -4 and coumarin backbone. (**B**) Molecular weight (MW), hydrogen bond donor (HBD), hydrogen bond acceptor (HBA), calculated octanol-water partition coefficient (cLogP), polar surface area (PSA), and predicted blood-brain barrier (BBB) score of these compounds. (**C**) Aβ aggregation inhibition of curcumin (as a positive control), coumarin, LM-031 and analogs (5–20 μM) by the thioflavin T assay (*n* = 3). To normalize, the relative thioflavin T fluorescence of Aβ_42_ without compound treatment was set at 100%. Shown below are the EC_50_ values. (**D**) Radical scavenging activity of kaempferol (as a positive control), coumarin, LM-031 and analogs (10–160 μM) on DPPH (*n* = 3). Shown below are the EC_50_ values.

We tested the coumarin derivative LM-031 and the analogs LMDS-1 to -4 (Figure 1A). According to their molecular weight (MW), hydrogen bond acceptor (HBA), hydrogen bond donor (HBD), and octanol-water partition coefficient (cLogP), these five compounds fulfill five criteria of Lipinski’s rule for predicting oral bioavailability [24] (Figure 1B). All five compounds were anticipated to diffuse over the BBB with a polar surface area (PSA) of smaller than 90 Å^2^ [25], which were similarly supported by an online BBB predictor [26] (Figure 1B).

Anti-amyloid and anti-oxidative stress are regarded as crucial AD therapeutic approaches. The thioflavin T fluorescence assay was applied to gauge the suppression of Aβ aggregation. Curcumin is known to reduce amyloid aggregation [27]. Both curcumin and coumarin were included for comparison. Half maximal effective concentration (EC_50_) values of curcumin, LM-031, LMDS-1 to -4 and coumarin to inhibit Aβ aggregation were: <5, 9, <5, 18, 14, <5 and 25 μM, respectively (Figure 1C). The free radical scavenging activity of the LM-031, analogs and coumarin was investigated using diphenylpicrylhydrazyl (DPPH) as a substrate. Kaempferol (a positive control [28]), LM-031, LMDS-1 to -4 and coumarin had EC_50_ values of 28, 104, 117, 146, 167, 116 and 179 μM, respectively (Figure 1D).

### Binding strength and conformation by docking computation

**Figure 2:**
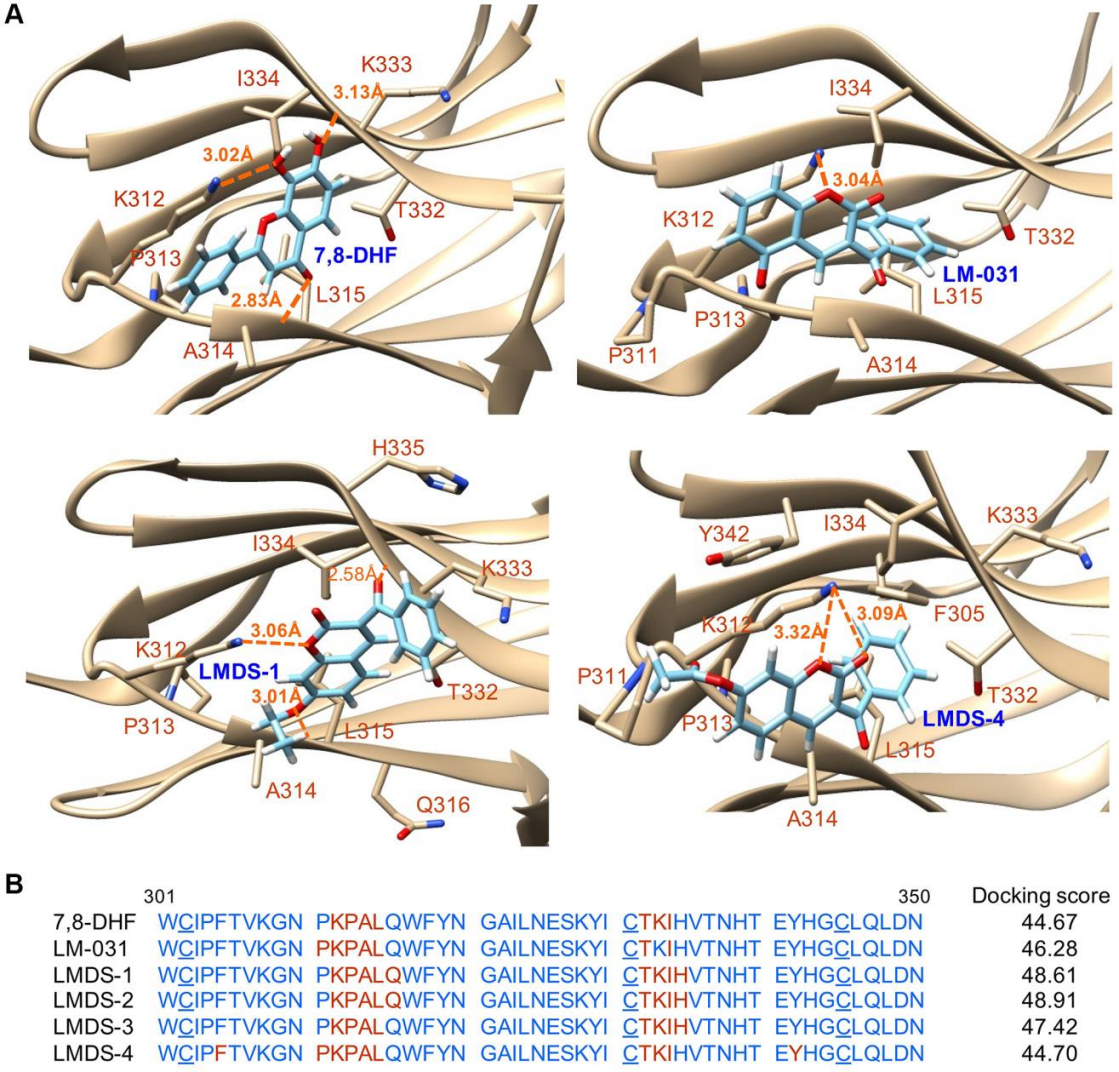
**Docking computations of 7,8-DHF, LM-031 and analogs.** (**A**) The docking conformations of 7,8-DHF (as a positive control), LM-031, LMDS-1 and LMDS-4 binding to extracellular d5 domain (the second immunoglobulin-like domain, residues 250–340) of TRKB receptor. The TRKB-d5 domain (ribbon structures) is colored in beige and the wire-frame structures denote the compounds. The labeled amino acids were within 10 Å radii of examined compounds. Carbon, oxygen, hydrogen and nitrogen atoms of compounds or side chains of surrounding amino acids are shown in light blue, red, white and blue, respectively. The dotted orange lines indicate hydrogen bond interactions between compounds and protein. (**B**) Amino acid residues 301–350 of d5 domain. The amino acids within 10 Å radii of examined compounds are colored in red; the cysteines involved in disulfide linkage are underlined. Shown on the right were docking scores of 7,8-DHF, LM-031 and analogs calculated by the GOLD program.

Figure 2A shows the binding conformations of 7,8-DHF, LM-031 and two representative LMDS compounds (LMDS-1 and -4) and Figure 2B shows the predicted binding strengths of these compounds. Of the tested compounds, the computations predicted that LMDS-1 and -2 were the top two compounds interacting with TRKB receptor. Note that the LMDS compounds have different binding modes in comparison with 7,8-DHF and LM-031. In addition, interestingly, all LMDS-1 to -3 have the same binding mode, 3 hydrogen bonds with backbone of L315 and I334, and side chain of K312. In contrast, LMDS-4 binds with d5 domain in different orientation with a lower binding strength compared with LMDS-1 to -3. It only has a bifurcated hydrogen bond with side chain of K312. These results suggest that bulky carboxylate group is not favorable functional group at the position of fused ring of LMDS compounds shown in Figure 1A.

### Aβ aggregation inhibition and reduction of Aβ-induced oxidative stress

**Figure 3:**
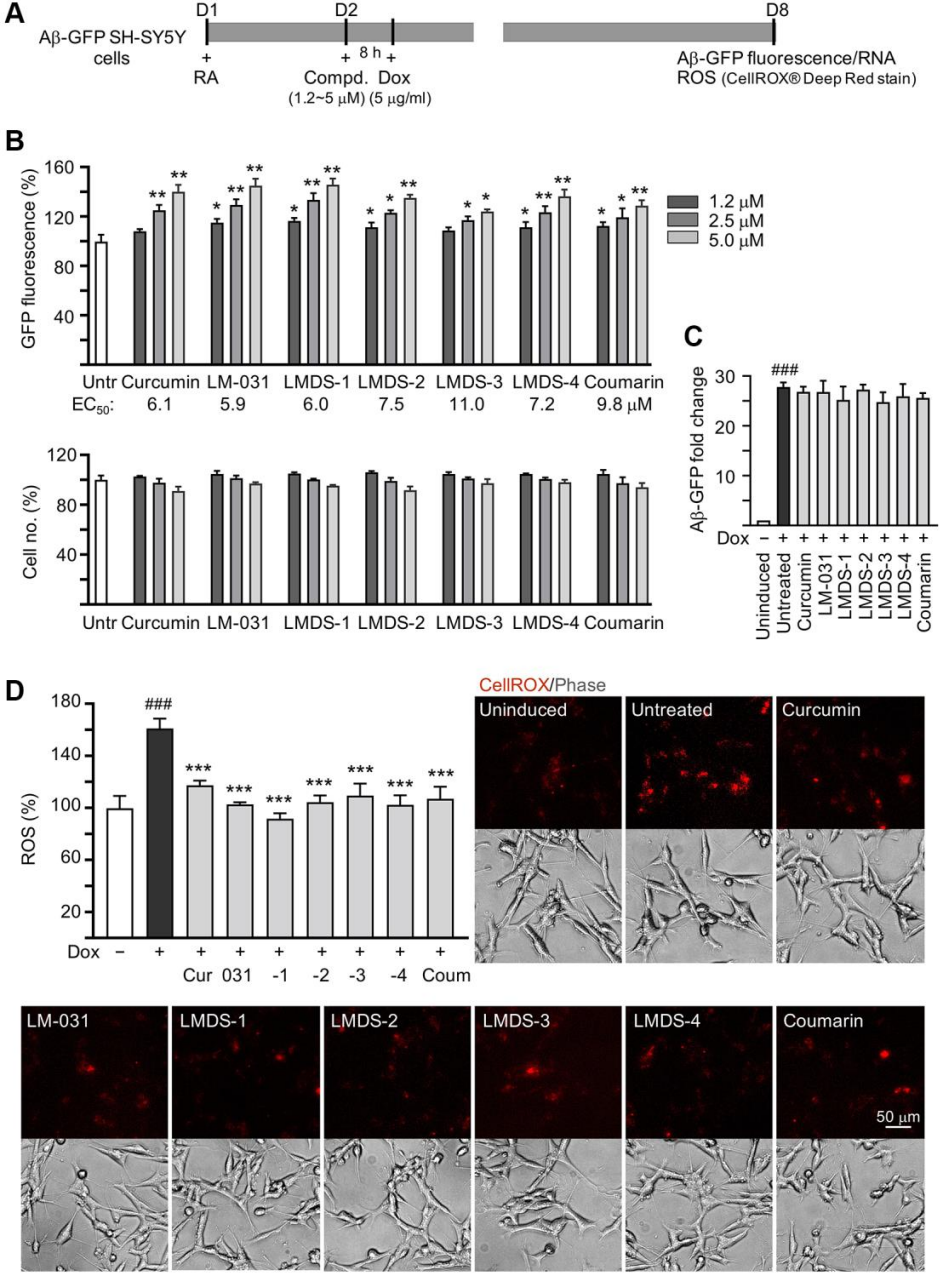
**Aβ aggregation and oxidative stress inhibitory effects of LM-031 and analogs in Aβ-GFP SH-SY5Y cells.** (**A**) Experimental flow chart. On day 1, cells were plated with retinoic acid (RA, 10 μM) added to the culture medium. On day 2, curcumin, coumarin, LM-031 or analogs (1.2–5 μM) was added to the cells for 8 h, followed by inducing Aβ-GFP expression with doxycycline (Dox, 5 μg/ml) for 6 days. On day 8, Aβ-GFP fluorescence, Aβ-GFP RNA and ROS (CellROX® Deep Red stain) were measured. (**B**) Assessment of GFP fluorescence with curcumin, coumarin, LM-031 or analogs (1.2–5 μM) treatment (*n* = 3), with EC_50_ values shown below. Shown underneath are cell number analyzed in each treatment. The relative GFP fluorescence/cell number of untreated cells (Untr) was normalized as 100%. (two-tailed Student’s *t* test; ^*^P < 0.05 and ^**^P < 0.01) (**C**) Aβ-GFP RNA of Aβ-GFP SH-SY5Y cells untreated or treated with curcumin, coumarin, LM-031 or analogs at 5 μM (*n* = 3). HPRT1 was used for normalization. (**D**) Images of CellROX® Deep Red stain (red) and ROS assay of Aβ-GFP cells uninduced, untreated, or treated with curcumin, coumarin, LM-031 or analogs at 5 μM (*n* = 3). The relative ROS of uninduced cells (Dox-) was normalized (100%). (**C**, **D**) *P* values: comparisons between induced (Dox+) vs. uninduced (Dox-) cells (^###^*P* < 0.001), or compound-treated vs. untreated (Dox+) cells (^***^P < 0.001). (one-way ANOVA with *post hoc* Tukey test).

Aβ-GFP SH-SY5Y cells [29] were used to examine Aβ aggregation inhibition of LM-031 and analogs (Figure 3A). The cell model uses GFP as a reporter to reflect the level of Aβ misfolding. The fast misfolding and formation of Aβ aggregates causes misfolding of fused GFP, thereby suppressing green fluorescence. Compounds that inhibit Aβ misfolding allow refolding of GFP monitored by an increase in green fluorescence on Aβ-GFP expressing cells [30]. Curcumin, which can modify the Aβ aggregation pathway and ameliorate Aβ-induced toxicity [31], was considered as a positive control. Coumarin was also included for comparison. The intensity of GFP fluorescence in cells pre-treated with 2.5–5 μM curcumin and 1.2–5 μM coumarin was significantly increased (113–140%, *P* = 0.037–0.001; cell viability: 101–91%) compared to the untreated cells (100%). Treatment with LM-031, LMDS-1, -2, -4 at 1.2–5 μM, or LMDS-3 at 2.5–5 μM significantly increased the intensity of green fluorescence (111–149%, *P* = 0.048–0.001; cell viability: 106–92%) (Figure 3B), while Aβ-GFP RNA level was not affected by LM-031 and analogs at 5 μM concentration (24.8–27.3 folds, *P* > 0.05) (Figure 3C). For Aβ aggregation inhibition, curcumin, LM-031, LMDS-1 to -4 and coumarin had EC_50_ values of 6.1, 5.9, 6.0, 7.5, 11.0, 7.2 and 9.8 μM, respectively. In an analysis of oxidative stress, the level of reactive oxygen species (ROS) increased significantly in Aβ-GFP-expressing SH-SY5Y cells (161%, *P* < 0.001), while treatments with curcumin, LM-031, analogs and coumarin at a concentration of 5 μM decreased the ROS level induced by induced Aβ expression (117–92%, *P* < 0.001) (Figure 3D). These results indicated that the LM-031 and analogs not only hindered the aggregation of amyloid, but also attenuated oxidative stress caused by Aβ overexpression.

### Neuroprotective activity of LM-031 and analogs

**Figure 4:**
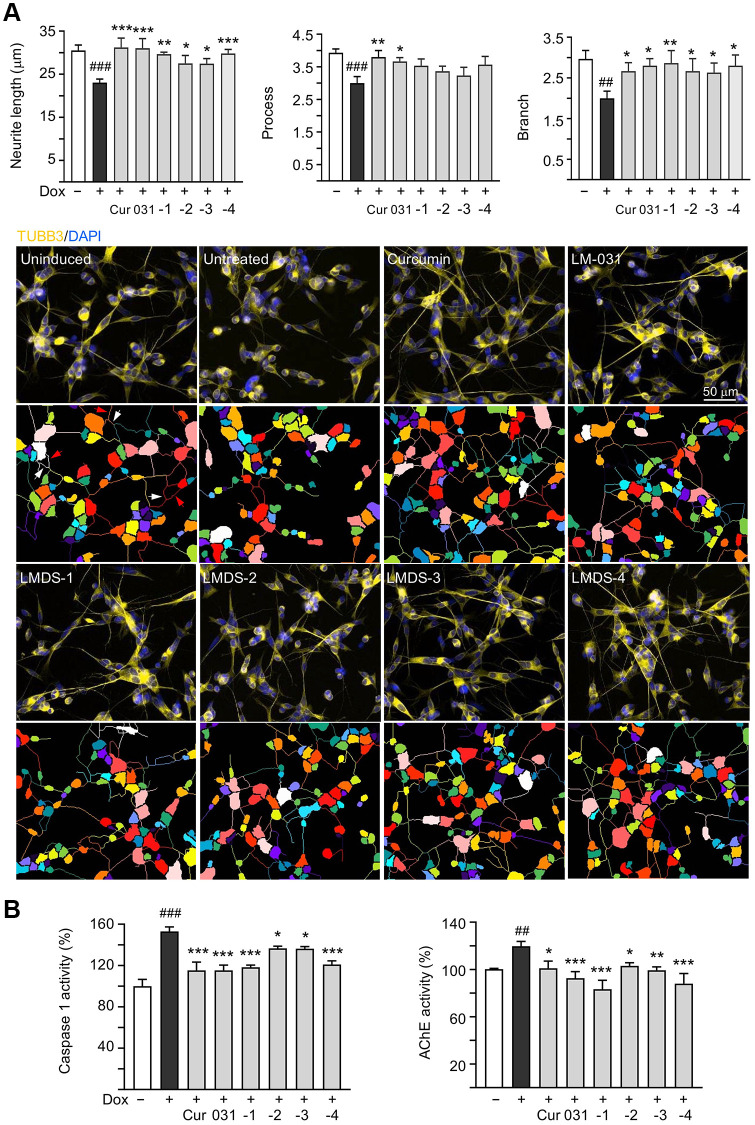
**Neuroprotective effects of LM-031 and analogs in Aβ-GFP SH-SY5Y cells.** (**A**) Neurite outgrowth (length, process and branch) assay of Aβ-GFP cells uninduced, untreated, or treated with curcumin, LM-031 or analogs at 5 μM (*n* = 3). Shown below are images of TUBB3 (yellow)-stained cells, with nuclei counterstained with DAPI (blue), and segmented images with multi-colored mask to assign each outgrowth to a cell body for quantification. In uninduced cells, processes and branches are indicated with red and white arrows, respectively. (**B**) Caspase 1 and AChE activity assays with curcumin, LM-031 or analogs (5 μM) treatment (*n* = 3). The relative caspase 1 or AChE activity of uninduced cells (Dox-) was normalized (100%). *P* values: comparisons between induced (Dox+) vs. uninduced (Dox-) cells (^##^*P* < 0.01 and ^###^*P* < 0.001), or compound-treated vs. untreated (Dox+) cells (^*^P < 0.05, ^**^P < 0.01, ^***^P < 0.001). (one-way ANOVA with *post hoc* Tukey test).

The neuroprotective efficacy of LM-031 and analogs, including acetylcholinesterase (AChE) and caspase 1 activity, and neurite outgrowth, were evaluated. Aβ overexpression significantly reduced length (from 30.6 μm to 23.1 μm, *P* = 0.002), process (from 3.9 to 3.0, *P* = 0.005) and branch (from 3.0 to 2.0, *P* = 0.002) of neurites. Respective curcumin, LM-031 or analogs (5 μM) treatment successfully increased neurite length (from 23.1 μm to 27.5–31.2 μm, *P* = 0.036–<0.001) and branch (from 2.0 to 2.7–2.9, *P* = 0.047–0.006), while rescue of process was evident only for curcumin and LM-031 (from 3.0 to 3.7–3.8, *P* = 0.011–0.002) (Figure 4A). AChE (120%, *P* = 0.007) and caspase 1 (153%, *P* < 0.001) activities were also considerably elevated by Aβ overexpression, whereas treatment with curcumin, LM-031 and analogs (5 μM) decreased AChE (from 120% to 103–83%; *P* = 0.027–<0.001) and caspase 1 (from 153% to 137–115%; *P* = 0.014–<0.001) activities in comparison to no treatment (Figure 4B).

### TRKB knockdown in Aβ-GFP-expressing SH-SY5Y cells

**Figure 5:**
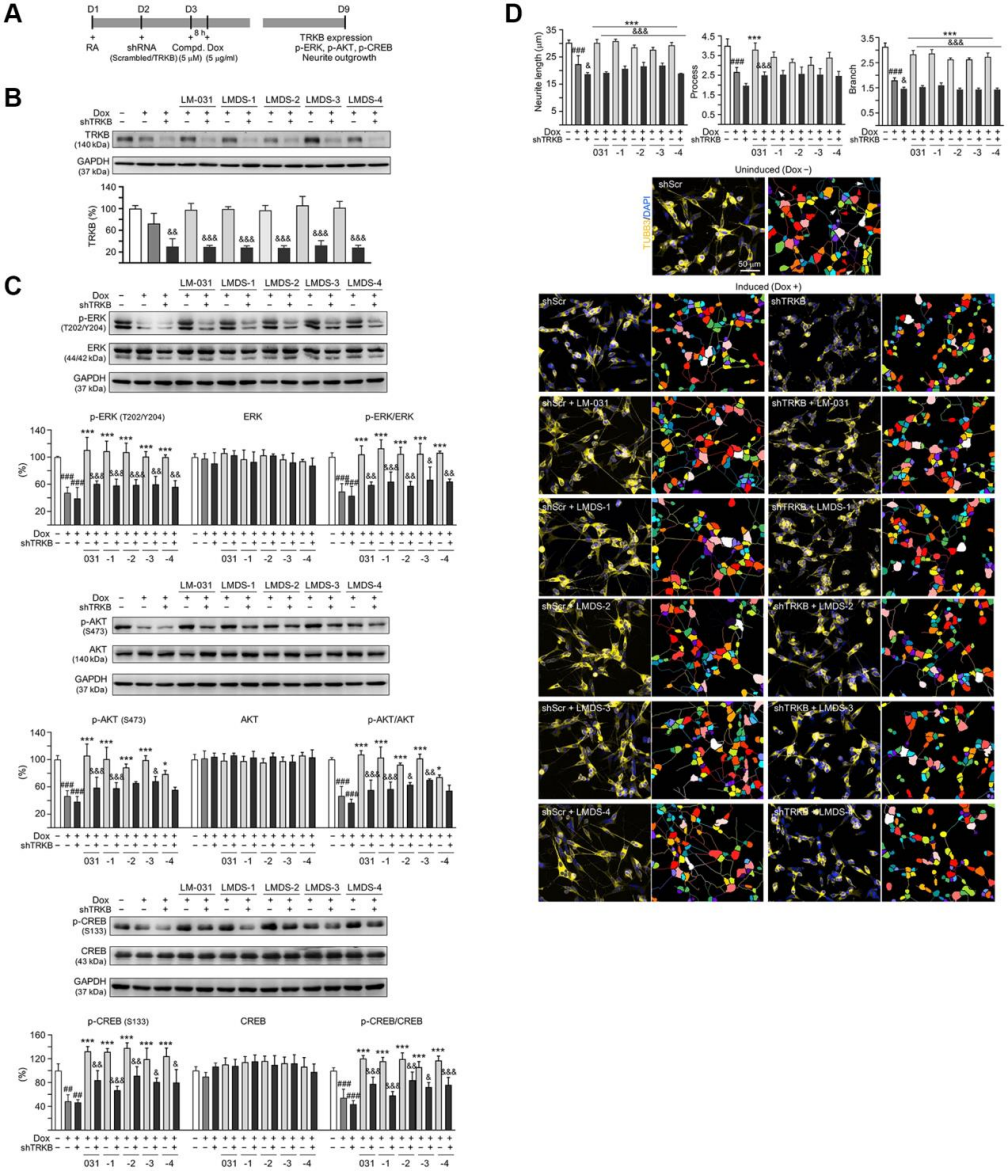
**TRKB RNA interference of Aβ-GFP SH-SY5Y cells.** (**A**) Experimental flow chart. On day 1, Aβ-GFP SH-SY5Y cells were plated with retinoic acid (RA; 10 μM). On day 2, the cells were infected with lentivirus-expressing TRKB-specific or scrambled shRNA. At 24 h post-infection, LM-031 or LMDS-1 to -4 (5 μM) was added to the cells for 8 h, followed by induction of Aβ-GFP expression (Dox, 5 μg/ml) for 6 days. On day 9, TRKB and neurite outgrowth analyses were performed. Western blot analysis of (**B**) TRKB, (**C**) p-ERK (T202/Y204), ERK, p-AKT (S473), AKT, p-CREB (S133), and CREB in compound-treated cells infected with TRKB-specific or scrambled shRNA-expressing lentivirus (*n* = 3). GAPDH was used as a loading control. To normalize, the relative protein level of uninduced cells was set at 100%. (**D**) Microscopic images and neurite outgrowth (length, process and branch) assay of Aβ-GFP-expressing cells with TRKB-specific or scrambled shRNA, and with or without LM-031 or analogs (5 μM) treatments (*n* = 3). TUBB3 staining (yellow) was used to quantify the extent of neurite outgrowth. Nuclei were counterstained with DAPI (blue). Also shown were segmented images with multi-colored mask to assign each outgrowth to a cell body for quantification. In uninduced cells, processes and branches are indicated with red and white arrows, respectively. *P* values: comparisons between induced vs. uninduced cells (^###^*P* < 0.001), compound-treated vs. untreated (induced) cells (^***^P < 0.001), or TRKB shRNA-treated vs. scrambled shRNA-treated cells (^&^*P* < 0.05, ^&&^*P* < 0.01, ^&&&^*P* < 0.001). (one-way ANOVA with a *post hoc* Tukey test).

Next, we used lentivirus-mediated shRNA targeting to knock down TRKB expression to assess the role of TRKB and downstream signaling (Figure 5A), since the investigated TRKB agonists had neuroprotective activity on Aβ-GFP-expressing SH-SY5Y cells. Aβ-GFP overexpression had no significant impact on TRKB expression in cells infected with control (scrambled) shRNA (72%; *P* > 0.05). TRKB expression was unaffected by treatment with LM-031 and analogs (97–106%; *P* > 0.05). In contrast, TRKB-specific shRNA decreased the amount of TRKB in Aβ-GFP-expressing cells without (from 75% to 30%, *P* = 0.002) or with (from 97–106% to 28–32%; *P* < 0.001) LM-031 and analogs treatment (Figure 5B).

In addition to examining the level of TRKB, the levels of downstream signaling effectors including ERK, AKT and CREB in TRKB-knockdown cells in response to treatment with LMDS compounds were also examined (Figure 5C). Aβ-GFP overexpression decreased the expression of p-ERK (47%, *P* < 0.001), p-AKT (46%, *P* < 0.001), and p-CREB (48%, *P* = 0.002) in cells infected with scrambled shRNA, and the reduction was not worsened by TRKB-specific shRNA infection (*P* > 0.05). Treatment with LM-031 or analogs rescued the reduced p-ERK (from 47% to 100–110%, *P* < 0.000), p-AKT (from 46% to 79–106%, *P* = 0.017–<0.000) and p-CREB (from 48% to 119–138%, *P* < 0.000) levels, and the rescue was blocked by TRKB-specific shRNA (p-ERK: 56–60%, *P* = 0.006–<0.001; p-AKT: 56–68%, *P* = 0.227–<0.001; p-CREB: 67–91%; *P* = 0.037–<0.001).

In the aforementioned TRKB-knockdown Aβ-GFP cells, the effects of LM-031 and analogs on increasing neurite outgrowth were assessed (Figure 5D). Overexpression of Aβ-GFP resulted in a significant decrease in length (from 30.2 μm to 22.4 μm), process (from 4.0 to 2.6), and branch (from 3.1 to 1.8) (*P* < 0.001) of neurite, and shRNA targeting TRKB furthermore decreased neurite length/branch to 18.7 μm/1.5 (*P* = 0.040–0.020). In Aβ-GFP cells, treatment with the LM-031 or analogs rescued the reduced neurite length (from 22.4 μm to 27.7–30.9 μm) and branch (from 1.8 to 2.6–2.9) (*P* < 0.001), and the rescue was blocked by shRNA targeting TRKB (length: 21.9–18.9 μm, branch: 1.6–1.4; *P* < 0.001). Neurite process was significantly increased only by LM-031 (from 2.6 to 3.8, *P* < 0.001), which was deterred by shRNA targeting TRKB (2.5, *P* < 0.001).

### Therapeutic targets of LMDS-1 and -2 in Aβ-GFP-expressing SH-SY5Y cells

**Figure 6:**
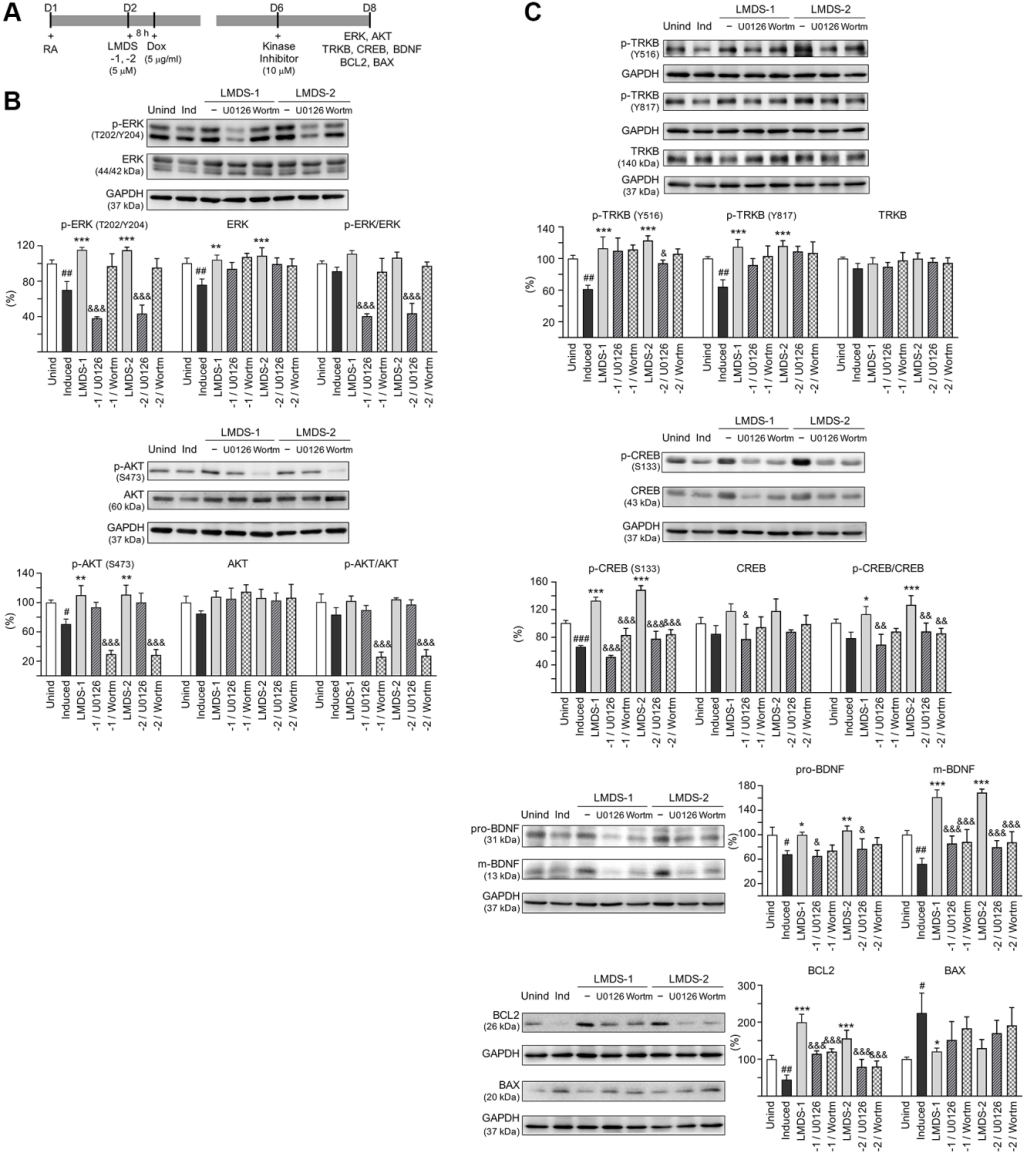
**Activation of ERK, AKT and CREB pathways downstream to TRKB in Aβ-GFP SH-SY5Y cells.** (**A**) Experimental flow chart. On day 1, cells were plated with retinoic acid (RA, 10 μM) added to the culture medium. On day 2, LMDS-1 or -2 (5 μM) was added to the cells for 8 h, followed by inducing Aβ-GFP expression with doxycycline (Dox, 5 μg/ml). Kinase inhibitors U0126 or wortmannin (10 μM) were added to the cells on day 6. On day 8, ERK, AKT, TRKB, CREB, BDNF, BCL2 and BAX levels were measured. (**B**) p-ERK (T202/Y204), ERK, p-AKT (S473), AKT, (**C**) p-TRKB (Y516 and Y817), TRKB, p-CREB (S133), CREB, BDNF (31/13 kDa), BCL2 and BAX levels analysed by immunoblot using GAPDH as a loading control (*n* = 3). To normalize, protein expression level in untreated cells was set at 100%. *P* values: comparisons between induced vs. uninduced cells (^#^*P* < 0.05, ^##^*P* < 0.01), compound-treated vs. untreated cells (^*^P < 0.05, ^**^P < 0.01, ^***^P < 0.001), or kinase inhibitor-treated vs. untreated cells (^&^*P* < 0.05, ^&&&^*P* < 0.001). (one-way ANOVA with a *post hoc* Tukey test).

The effects of LMDS-1 and LMDS-2 on expression levels of ERK, AKT and downstream targets of TRKB were examined by applying ERK inhibitor U0126 or PI3K inhibitor wortmannin (10 μM) to LMDS-1/2-treated SH-SY5Y cells expressing Aβ-GFP (Figure 6A). Overexpression of Aβ-GFP down-regulated p-ERK and p-AKT (70–71%, *P* = 0.004–0.007), LMDS-1 and -2 treatment rescued the reduction (110–115%, *P* < 0.001), whereas U0126 treatment lowered the elevation of p-ERK (from 115% to 38–43%, *P* < 0.001) and wortmannin treatment lowered the elevation of p-AKT (from 110–111% to 30–29%, *P* < 0.001) (Figure 6B). Additionally, induced expression of Aβ-GFP reduced p-TRKB Y516 and Y817 (61–64%, *P* = 0.004–0.001), p-CREB (66%, *P* < 0.001), pro- and m-BDNF (68–52%, *P* = 0.022–0.005) and BCL2 (44%, *P* = 0.009), and increased BCL2 associated X, apoptosis regulator (BAX) (225%, *P* = 0.012). In contrast, treatment with LMDS-1 and -2 increased p-TRKB Y516 and Y817 (113–123%, *P* < 0.001), p-CREB (127–142%, *P* < 0.001), pro- and m-BDNF (100–169%, *P* = 0.025–<0.001) and BCL2 (156–199%, *P* < 0.001), and reduced BAX (121–130%, *P* = 0.045–0.080). Treatment with U0126 or wortmannin attenuated the increase in p-CREB (52–84%, *P* < 0.001), pro- and m-BDNF (65–88%, *P* = 0.198–<0.001) and BCL2 (80–121%, *P* < 0.001), and reduced the decrease in BAX (152–191%, *P* > 0.05) (Figure 6C).

### Comparative effects of LMDS-1, -2 and BDNF on TRKB signaling

**Figure 7:**
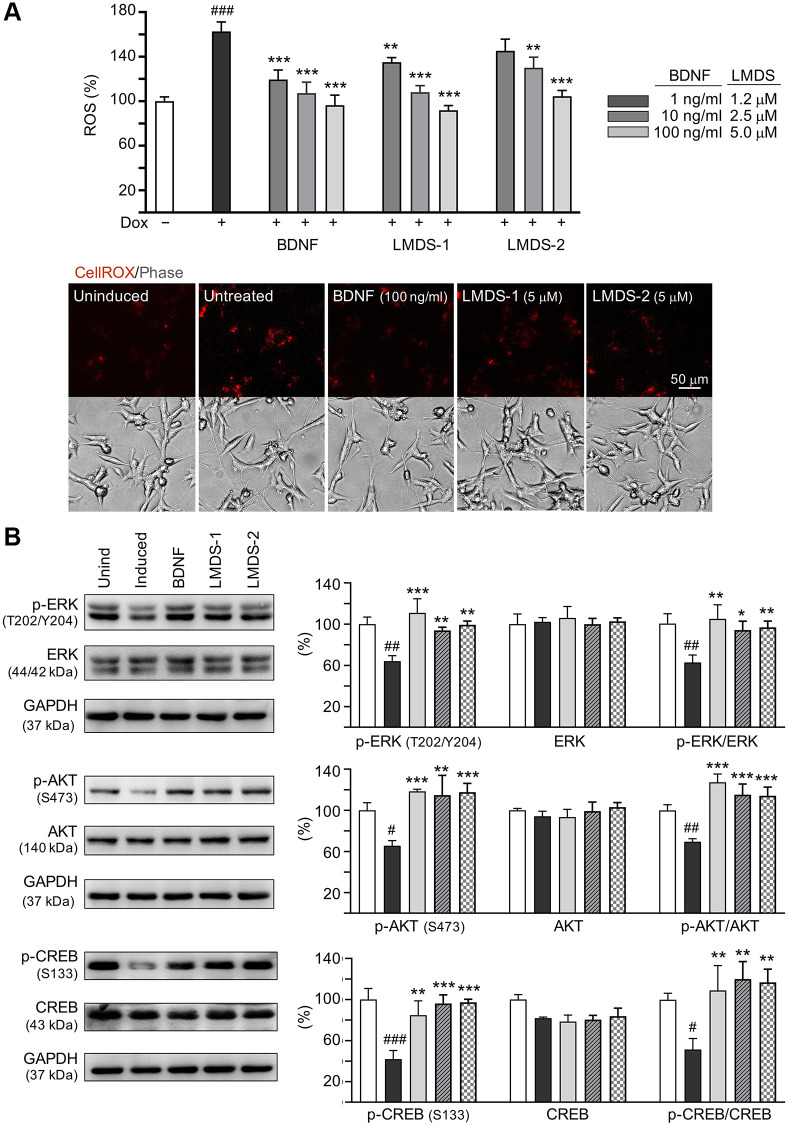
**Comparison of BDNF’s and LMDS compound’s signaling activation.** (**A**) Assessment of ROS in Aβ-GFP cells uninduced, untreated, treated with treated with BDNF at 1–100 ng/ml, or LMDS-1, -2 at 1.2–5 μM (*n* = 3). The relative ROS of uninduced cells (Dox-) was normalized (100%). Shown below are images of CellROX® Deep Red stain (red). (**B**) Assessment of p-ERK (T202/Y204), ERK, p-AKT (S473), AKT, p-CREB (S133) and CREB levels with LMDS-1, -2 (5 μM) or BDNF (100 ng/ml) treatment by immunoblot using GAPDH as a loading control (*n* = 3). To normalize, protein expression level in untreated cells was set at 100%. *P* values: comparisons between induced vs. uninduced cells (^#^*P* < 0.05, ^##^*P* < 0.01), or compound-treated vs. untreated cells (^*^P < 0.05, ^**^P < 0.01, ^***^P < 0.001). (one-way ANOVA with a *post hoc* Tukey test).

As LMDS-1 and -2 target ERK, AKT and downstream CREB, effects of BDNF and LMDS compounds on TRKB signaling were compared. Oxidative stress attenuation was first evaluated by treating Aβ-GFP cells with BDNF at 1–100 ng/ml concentration or LMDS-1, -2 at 1.2–5 μM concentration (Figure 7A). The ROS level induced by Aβ overexpression was successfully decreased by BDNF at 1–100 ng/ml, LMDS-1 at 1.2–5 μM, and LMDS-2 at 2.5–5 μM (from 163% to 135–92%, *P* = 0.007–<0.001). BDNF at 100 ng/ml and LMDS-1, -2 at 5 μM were then selected to compare the efficacy in TRKB signaling (Figure 7B). Overexpression of Aβ-GFP down-regulated p-ERK (64%, *P* = 0.001), p-AKT (65%, *P* = 0.014) and p-CREB (42%, *P* < 0.001), and BDNF, LMDS-1 and -2 treatment rescued the reduction (p-ERK: 94–111%, *P* = 0.005–<0.001; p-AKT: 115–118%, *P* = 0.001–<0.001; p-CREB: 85–97%, *P* = 0.002–<0.001). No significant differences in p-ERK, p-AKT and p-CREB were detected between BDNF and LMDS-1/2 groups (*P* > 0.05).

### BBB permeability of LM-031 and analogs

As of now, the PAMPA model has been proposed to analyze membrane permeability, including BBB [32, 33]. The *in vitro* BBB permeability of LM-031 and analogs was then estimated using the PAMPA-BBB approach. Quality controls, including high permeability bupropion [33], low permeability piroxicam [32], and integrity marker lucifer yellow, were included for comparison. Bupropion and piroxicam, which represent high (> 4 × 10^−6^ cm/s) and low (< 2 × 10^−6^ cm/s) BBB permeable controls, had effective permeability (P_e_) values of 17.06 and 1.65 (10^−6^ cm/s), respectively (Table 1). Well-accepted membrane integrity (below the 0.1% cut-off) was demonstrated by the unnoticeable transport of Lucifer yellow. The P_e_ values of LM-031, LMDS-1, -2 and -3 were 4.80 ± 0.12 [34], 14.88 ± 3.61, 3.81 ± 0.13 and 11.83 ± 4.11 (10^−6^ cm/s) respectively, suggesting to be categorized as BBB permeable (P_e_ > 2 ×10^−6^ cm/s) in PAMPA-BBB measurement. Due to the low mass recovery (12.7%), the P_e_ value of LMDS-4 was not determined.

**Table 1:** Permeability of LM-031, analogs and QC compound by PAMPA-BBB method.

**Compound name**	**Measured P_e_ (10^−6^ cm/s) or % transport**	**PAMPA-BBB classification^*a*^**
LM-031	4.80 ± 0.12	BBB^+^ (high)
LMDS-1	14.88 ± 3.61	BBB^+^ (high)
LMDS-2	3.81 ± 0.13	BBB^+^ (moderate)
LMDS-3	11.83 ± 4.11	BBB^+^ (high)
LMDS-4	–	Not determined (due to low mass recovery)
Bupropion	17.06 ± 1.09	High marker [33]
Piroxicam	1.65 ± 0.17	Low marker [32]
Lucifer yellow	0.00 (% Transport)	Integrity marker

^*a*^Suggested BBB permeability classification: P_e_ > 4 × 10^−6^ cm/s (high), 4 > P_e_ > 2 × 10^−6^ cm/s (moderate), and P_e_ < 2 × 10^−6^ cm/s (low).

## DISCUSSION

There are several reasons for identifying small molecules acting as novel potential TRKB agonists for AD therapeutic uses. First, there is no available treatment currently to cure AD or to halt its progression. Second, substantial evidence has shown that decreased BDNF and impaired TRKB pathways, including ERK, CREB and PI3K-AKT, contribute to neurodegeneration in AD [13–17]. Third, agents enhancing BDNF expression or TRKB agonists provide beneficial effects to AD [35–37]. Fourth, BDNF hardly penetrates BBB into brain [21]. Thus, identifying TRKB agonists with good BBB permeability may provide a therapeutic strategy for AD. In the present study, we identified novel potential TRKB agonists with good bioavailability (Figure 1). This is supported by the calculated values of MW, HBD, HBA and cLogP of these compounds because they satisfy the Lipinski’s criteria [24]. Furthermore, PSA of less than 90 Å^2^ [25] and predicted BBB scores of greater than threshold 0.02 [26] support a good BBB permeability of these compounds (Figure 1). The binding scores predicted by docking computations suggest these compounds have a good affinity to d5-domain of TRKB (Figure 2), providing evidence of their potential as TRKB agonists.

LM-031 and LMDS-1 to -4 significantly reduced ROS production (Figure 3) and promoted neurite outgrowth (Figure 4) in induced Aβ-GFP SH-SY5Y cells. TRKB signaling is a key factor regulating neuron survival, differentiation, dendritic and axonal growth and branching, as well synaptic plasticity [38–40]. In addition, TRKB agonist 7,8-DHF displayed anti-oxidative, anti-inflammatory, and anti-apoptotic effects to protect against cerebral injury [41]. Consistent with these previous reports, our results showed that LM-031 and analogs stimulated neurite outgrowth and branching by activating TRKB signaling, and knock-down of TRKB significantly attenuated the neurite outgrowth promoting effects (Figure 5).

In addition to targeting TRKB, neurotrophic receptor tyrosine kinase 1 (TRKA) agonist D3 have been shown to provide beneficial effects to activate TRKA-related signaling cascades and enhance cholinergic neurotransmission in transgenic mice overexpressing human APP with KM670/671NL and V717F mutations [42]. Targeting neurotrophic receptor tyrosine kinase 3 (TRKC) signaling may also be an effective approach to rescue impaired cholinergic function in AD [43]. Our study results demonstrate that LMDS compounds specific for TRKB binding enhance neurite outgrowth by activating signaling downstream of TRKB. Whether TRK receptor isoforms TRKA and TRKC could potentially be activated by LMDS compounds remains to be determined. In addition, future *in vitro* biding assay should be performed to provide evidence of LMDS compounds binding to extracellular domain of TRKB [22] to show their specificity of TRKB binding.

Once TRKB is activated, three major signaling cascades, PLC-γ1, ERK and PI3K-AKT, ensue [44]. CREB can be phosphorylated at S133 by both p-ERK and p-AKT, and p-CREB (S133) can further enhance BDNF expression. We examined if LM-031, LMDS-1 and -2 would act on TRKB signaling pathways, including ERK-CREB and PI3K-AKT-CREB. As shown in Figure 6, ERK inhibitor U0126 and PI3K inhibitor wortmannin, respectively, decreased p-CREB and mature BDNF in SH-SY5Y cells expressing Aβ-GFP, suggesting that LM-031, LMDS-1 and -2 exerted their neuroprotection effects via enhancing PI3K-AKT-CREB and ERK-CREB pathways. CREB phosphorylation up-regulates cAMP responsive element-mediated genes, including BDNF and BCL2, which provides neuroprotective effects against apoptosis [45, 46]. BCL2 was enhanced by LMDS-1 and -2 and BAX reduced by LMDS-1, which effects were diminished by ERK inhibitor or PI3K inhibitor, further implicating that ERK, PI3K-AKT and CREB pathways contribute to neuroprotection in the Aβ-GFP model.

Several known small molecules or compounds such as catalpol, cystamine, rosmarinic acid and wogonin could increase BDNF expression, but their role as TRKB agonists are not validated [47–50]. Up to now, a few TRKB agonists have been developed to treat neurodegenerative models. Among them, 7,8-DHF demonstrates the features of a potent TRKB agonist and the effects of preventing Aβ deposition, deficits of hippocampal synapses and memory impairment in 5×FAD (APP K670N, M671L, I716V and V717I mutations along with PS1 M146L and L286V mutations) [51] or Tg2576 (APP K670N and M671L mutations) [52] AD mice. 7,8-DHF also elevates cellular glutathione levels and diminishes ROS stress caused by glutamate in HT-22 cells [53] and protects PC12 cells from cytotoxicity induced by 6-hydroxydopamine through increasing superoxide dismutase and decreasing malondialdehyde [54]. It also suppresses the accumulation of α-synuclein and oxidative stress via activating TRKB [55]. Similar to 7,8-DHF, the LMDS-1 and -2 also significantly reduced oxidative stress in Aβ-GFP cells. 7,8-DHF has a high affinity with TRKB [56, 57]. LM22A-4 is also a TRKB agonist, which has shown TRKB-activating and neurodegeneration-preventing effects in rodents, but LM22A-4 has a poor BBB penetration ability [58]. Similar to 7,8-DHF, LMDS-1 and -2, respectively displayed a good BBB permeability as shown by PAMPA-BBB measurement (Table 1), suggesting their potentials to treat neurodegenerative diseases. Application of LMDS-1 and -2 to animal models are warranted to confirm the neuroprotection effects.

## CONCLUSIONS

In this study, we identified LM-031 analogous compounds LMDS-1 and -2 which may serve as the potential TRKB agonists to treat AD. This is supported by our experimental results demonstrating TRKB signaling-activating, aggregation-inhibitory, oxidative stress- and caspase 1-reducing, and neurite outgrowth-promoting effects in Aβ-GFP SH-SY5Y cells. Pursuit of analogous compounds resembled to the identified compounds should be of interest in the future.

## MATERIALS AND METHODS

### Test compounds and BDNF

In-house LM-031 activating the CREB-dependent survival and anti-apoptosis pathway was prepared as described [23]. To search for LM-031 analogous compounds, InterBioScreen Ltd. (https://www.ibscreen.com/), ChEMBL (https://www.ebi.ac.uk/chembl/) and ZINC (http://zinc15.docking.org/) databases were screened using compound similarity search software tools. Four top-scoring compounds LMDS-1 to -4 were selected and obtained from Enamine (Kyiv, Ukraine). In cell culture media, these five compounds had no solubility problems all the way to 100 μM. The following items were bought from Sigma-Aldrich Co. (St. Louis, MO, USA): curcumin (control for monitoring Aβ folding), kaempferol (antioxidant control in DPPH assay), coumarin (backbone of LM-031 and analogs) and BDNF.

### Bioavailability and BBB permeation prediction

Using Internet software ChemDraw (http://www.perkinelmer.com/tw/category/chemdraw), the following properties of LM-031 and its analogs were computed: MW, HBD, HBA, cLogP, and PSA. Additionally, BBB permeation scores were derived utilizing the BBB prediction server Online BBB Predictor (https://www.cbligand.org/BBB/) [26].

### Anti-amyloid and antioxidant assays

Thioflavin T is widely used to visualize and quantify the presence of misfolded amyloid protein aggregates *in vitro* [59]. The Aβ amyloid inhibiting potential of curcumin, LM-031 and analogs (5–20 μM) were assessed by using Aβ42 peptide (AnaSpec, Fremont, CA, USA) upon binding to thioflavin T as stated [60]. In addition, DPPH radical (Sigma-Aldrich) [61] was used to assay the free radical scavenging capacity of kaempferol, LM-031 and analogs (10–160 μM) as stated [60].

### Docking computation

Docking calculations for LM-031 and analogs were carried out using the GOLD docking program [62, 63]. The computational protocol was similar to our previous study [64]. Briefly, protein structure of domain d5 of TRKB (pdb code: 1HCF) [65] was used for docking compounds. The d5 domain determined the specificity of neurotrophin receptors experimentally [66]. Binding site of small molecule agonists has been predicted by a previous computation [67]. To assure convergence of the computed findings, the numbers of operations of 10,000, 20,000, 40,000 and 80,000 were carried out in the current docking computations of LM-031 and analogs. For comparison, 7,8-DHF, a bioactive TRKB agonist [22], was also added to the computation.

### Cells and culture

Human neuroblastoma SH-SY5Y-derived Aβ-GFP cells were maintained as stated [29]. The recombinant Aβ-GFP protein has a 12 amino acid-containing linker between the fused Aβ and GFP. Doxycycline (5 μg/ml; Sigma-Aldrich) was used to induce Aβ-GFP expression.

### Aβ folding reporter fluorescence and oxidative stress analyses

Neuronal differentiation of Aβ-GFP SH-SY5Y cells was induced by retinoic acid (10 μM; Sigma-Aldrich) [68]. Cells on 96-well plate (2.5 × 10^4^/well) were seeded on day 1, pre-treated with test compounds (1.2–5 μM) plus induced Aβ-GFP expression (5 μg/ml doxycycline) on day 2, and stained with Hoechst 33342 (0.1 μg/ml; Sigma-Aldrich) on day 8 as stated [60]. Cell images were then captured at 482 nm excitation/536 nm emission wavelengths (ImageXpress Micro Confocal high-content system) and analyzed (MetaXpress image acquisition and analysis software) (Molecular Devices, Sunnyvale, CA, USA). In addition, ROS was quantified on day 8 following CellROX Deep Red stain (5 μM; Molecular Probes, Waltham, MA, USA) using the high-content system with excitation/emission wavelengths of 644/665 nm.

### Real-time PCR analysis

Total RNA was extracted, converted to cDNA, and the expressed Aβ-GFP RNA was quantified as stated [69]. The formula 2^ΔCt^, ΔCT = C_T_ (HPRT1) - C_T_ (EGFP), where CT stands for cycle threshold, was used to compute fold change.

### Neurite outgrowth analysis

As stated, Aβ-GFP SH-SY5Y cells were seeded (6 × 10^4^/24-well) with retinoic acid addition on day 1, treated with tested compounds (5 μM) plus induced Aβ-GFP expression (5 μg/ml doxycycline) on day 2, and stained with TUBB3 (tubulin beta 3 class III) primary antibody (1:1000; Covance #MMS-435P, Princeton, NJ, USA), goat anti-rabbit Alexa Fluor^®^ 555 secondary antibody (1:1000; Thermo Fisher Scientific #A27039), and DAPI (4′-6-diamidino-2-phenylindole, 0.1 μg/ml; Sigma-Aldrich) on day 8 [60]. Neuronal pictures were taken using the high-content system and analyzed for neurite length (μm), process (number of neurites protruding from neuronal cell body) and branch (number of neurites extending from process) (MetaXpress neurite outgrowth application module; Molecular Devices). In each of three independent experiments, roughly 6000 cells from each sample were examined.

### Caspase 1 and AChE activity assays

Cells on 6-well plate (5 × 10^5^/well) were subjected to the retinoic acid, test compound and doxycycline treatments as stated. Cells were collected on day 8 and cell lysates prepared for caspase 1 (BioVision, Milpitas, CA, USA) and AChE (Sigma-Aldrich) activity measurement [60].

### TRKB RNA interference

To knock down TRKB expression in Aβ-GFP SH-SY5Y cells, lentiviral short hairpin RNA (shRNA) targeting TRKB and control (scrambled) were used [69]. Cells were plated in the presence of retinoic acid (5 × 10^5^/6-well for protein analysis or 6 × 10^4^/24-well for neurite outgrowth analysis) on day 1, infected with lentivirus on day 2, pretreated with test compound (5 μM) plus inducing Aβ-GFP expression on day 3, and collected for neurite outgrowth or TRKB protein analysis on day 9 as stated [69].

### ERK/PI3K kinase inhibitor treatment

Cells were plated in the presence of retinoic acid (5 × 10^5^/6-well) on day 1, treated with tested compounds (5 μM) plus induced Aβ-GFP expression with doxycycline (5μg/ml) on day 2, as stated. Kinase inhibitors U0126 (an inhibitor of ERK) or wortmannin (an inhibitor of PI3K) (LC Laboratories, Woburn, MA, USA) (10 μM) were added on day 6. The cells were collected on day 8 for BDNF, BCL2, BAX, total/phosphorylated TRKB, ERK, AKT, and CREB protein analysis as previously stated.

### Immunoblot analysis

Total proteins from Aβ-GFP SH-SY5Y cells were prepared, quantified, electrophoretically separated and blotted as stated [60]. Following blocking, the membrane was probed with antibody against BDNF (1:500; Santa Cruz Biotechnology #sc-546, Santa Cruz, CA, USA), BCL2 (1:200; Santa Cruz Biotechnology #sc-7382), BAX (1:200; Santa Cruz Biotechnology #sc-7480), TRKB (1:500; Cell Signaling Technology #4603, Danvers, MA, USA), p-TRKB (Y516) (1:200; GeneTex #GTX32230, Irvine, CA, USA), p-TRKB (Y816) (1:1000; Millipore #ABN1381, Billerica, MA, USA), ERK (1:1000; Cell Signaling Technology #9102), p-ERK (T202/Y204) (1:1000; Cell Signaling Technology #9101), AKT (1:1000; Abcam #ab126811, Cambridge, CB, UK), p-AKT (S473) (1:1000; Cell Signaling Technology #4060), CREB (1:500; Santa Cruz Biotechnology #sc-186), p-CREB (S133) (1:1000; Millipore #06-519), or loading control GAPDH (glyceraldehyde-3-phosphate dehydrogenase) (1:1000; MDBio #30000002, Taipei, Taiwan). The immune complexes were detected as stated [60].

### PAMPA to assess BBB permeability

The permeability of LMDS-1 and -2 was determined by a PAMPA-BBB assay. Firstly, 300 μl of LMDS-1 or -2 (1 μM) solution were added to the donor well (Millipore). Bupropion (a high permeability marker), piroxicam (a low permeability marker), and lucifer yellow (an integrity marker) (Sigma-Aldrich) were used as quality control (QC) compounds for comparison. The sandwich with aqueous donor on the bottom, artificial lipid membrane (PVDF filter coated with porcine polar brain lipid) in the middle, and aqueous acceptor (5% DMSO in PBS) on the top was assembled as stated [69]. Each compound was tested in triplicate. After incubation for 18 h at the ambient temperature, the PAMPA sandwich plate was separated. The concentrations of test and QC compounds in the donor and acceptor wells were measured as stated [69], and the effective permeability coefficient (P_e_) was computed [70].

### Statistical analysis

The mean ± standard deviation from three independent experiments are reported as the data. As appropriate, a two-tailed Student’s *t* test or one-way ANOVA (analysis of variance) with a *post hoc* Tukey test were used to assess group differences. *P* values < 0.05 indicated statistical significance.

## References

[1] Goedert M, Sisodia SS, Price DL. Neurofibrillary tangles and beta-amyloid deposits in Alzheimer's disease. Curr Opin Neurobiol. 1991; 1:441–7. 10.1016/0959-4388(91)90067-h 1821689

[2] Hardy J. Amyloid, the presenilins and Alzheimer's disease. Trends Neurosci. 1997; 20:154–9. 10.1016/s0166-2236(96)01030-2 9106355

[3] Hardy J, Allsop D. Amyloid deposition as the central event in the aetiology of Alzheimer's disease. Trends Pharmacol Sci. 1991; 12:383–8. 10.1016/0165-6147(91)90609-v 1763432

[4] Roher AE, Lowenson JD, Clarke S, Woods AS, Cotter RJ, Gowing E, Ball MJ. beta-Amyloid-(1-42) is a major component of cerebrovascular amyloid deposits: implications for the pathology of Alzheimer disease. Proc Natl Acad Sci U S A. 1993; 90:10836–40. 10.1073/pnas.90.22.10836 8248178 PMC47873

[5] Koo EH, Lansbury PT Jr, Kelly JW. Amyloid diseases: abnormal protein aggregation in neurodegeneration. Proc Natl Acad Sci U S A. 1999; 96:9989–90. 10.1073/pnas.96.18.9989 10468546 PMC33726

[6] Small DH, Mok SS, Bornstein JC. Alzheimer's disease and Abeta toxicity: from top to bottom. Nat Rev Neurosci. 2001; 2:595–8. 10.1038/35086072 11484003

[7] Butterfield DA, Swomley AM, Sultana R. Amyloid β-peptide (1-42)-induced oxidative stress in Alzheimer disease: importance in disease pathogenesis and progression. Antioxid Redox Signal. 2013; 19:823–35. 10.1089/ars.2012.5027 23249141 PMC3749710

[8] Liu L, Chan C. The role of inflammasome in Alzheimer's disease. Ageing Res Rev. 2014; 15:6–15. 10.1016/j.arr.2013.12.007 24561250 PMC4029867

[9] Lu B, Nagappan G, Guan X, Nathan PJ, Wren P. BDNF-based synaptic repair as a disease-modifying strategy for neurodegenerative diseases. Nat Rev Neurosci. 2013; 14:401–16. 10.1038/nrn3505 23674053

[10] Islam O, Loo TX, Heese K. Brain-derived neurotrophic factor (BDNF) has proliferative effects on neural stem cells through the truncated TRK-B receptor, MAP kinase, AKT, and STAT-3 signaling pathways. Curr Neurovasc Res. 2009; 6:42–53. 10.2174/156720209787466028 19355925

[11] Murray PS, Holmes PV. An overview of brain-derived neurotrophic factor and implications for excitotoxic vulnerability in the hippocampus. Int J Pept. 2011; 2011:654085. 10.1155/2011/654085 21966294 PMC3182334

[12] Zuccato C, Cattaneo E. Brain-derived neurotrophic factor in neurodegenerative diseases. Nat Rev Neurol. 2009; 5:311–22. 10.1038/nrneurol.2009.54 19498435

[13] Phillips HS, Hains JM, Armanini M, Laramee GR, Johnson SA, Winslow JW. BDNF mRNA is decreased in the hippocampus of individuals with Alzheimer's disease. Neuron. 1991; 7:695–702. 10.1016/0896-6273(91)90273-3 1742020

[14] Peng S, Wuu J, Mufson EJ, Fahnestock M. Precursor form of brain-derived neurotrophic factor and mature brain-derived neurotrophic factor are decreased in the pre-clinical stages of Alzheimer's disease. J Neurochem. 2005; 93:1412–21. 10.1111/j.1471-4159.2005.03135.x 15935057

[15] Rosa E, Fahnestock M. CREB expression mediates amyloid β-induced basal BDNF downregulation. Neurobiol Aging. 2015; 36:2406–13. 10.1016/j.neurobiolaging.2015.04.014 26025137

[16] Vitolo OV, Sant'Angelo A, Costanzo V, Battaglia F, Arancio O, Shelanski M. Amyloid beta -peptide inhibition of the PKA/CREB pathway and long-term potentiation: reversibility by drugs that enhance cAMP signaling. Proc Natl Acad Sci U S A. 2002; 99:13217–21. 10.1073/pnas.172504199 12244210 PMC130613

[17] Tong L, Balazs R, Thornton PL, Cotman CW. Beta-amyloid peptide at sublethal concentrations downregulates brain-derived neurotrophic factor functions in cultured cortical neurons. J Neurosci. 2004; 24:6799–809. 10.1523/JNEUROSCI.5463-03.2004 15282285 PMC6729714

[18] Han K, Jia N, Li J, Yang L, Min LQ. Chronic caffeine treatment reverses memory impairment and the expression of brain BNDF and TrkB in the PS1/APP double transgenic mouse model of Alzheimer's disease. Mol Med Rep. 2013; 8:737–40. 10.3892/mmr.2013.1601 23900282 PMC3782531

[19] Fukumoto K, Mizoguchi H, Takeuchi H, Horiuchi H, Kawanokuchi J, Jin S, Mizuno T, Suzumura A. Fingolimod increases brain-derived neurotrophic factor levels and ameliorates amyloid β-induced memory impairment. Behav Brain Res. 2014; 268:88–93. 10.1016/j.bbr.2014.03.046 24713151

[20] Shin MK, Kim HG, Baek SH, Jung WR, Park DI, Park JS, Jo DG, Kim KL. Neuropep-1 ameliorates learning and memory deficits in an Alzheimer's disease mouse model, increases brain-derived neurotrophic factor expression in the brain, and causes reduction of amyloid beta plaques. Neurobiol Aging. 2014; 35:990–1001. 10.1016/j.neurobiolaging.2013.10.091 24268884

[21] Dittrich F, Ochs G, Grosse-Wilde A, Berweiler U, Yan Q, Miller JA, Toyka KV, Sendtner M. Pharmacokinetics of intrathecally applied BDNF and effects on spinal motoneurons. Exp Neurol. 1996; 141:225–39. 10.1006/exnr.1996.0157 8812156

[22] Jang SW, Liu X, Yepes M, Shepherd KR, Miller GW, Liu Y, Wilson WD, Xiao G, Blanchi B, Sun YE, Ye K. A selective TrkB agonist with potent neurotrophic activities by 7,8-dihydroxyflavone. Proc Natl Acad Sci U S A. 2010; 107:2687–92. 10.1073/pnas.0913572107 20133810 PMC2823863

[23] Lee SY, Chiu YJ, Yang SM, Chen CM, Huang CC, Lee-Chen GJ, Lin W, Chang KH. Novel synthetic chalcone-coumarin hybrid for Aβ aggregation reduction, antioxidation, and neuroprotection. CNS Neurosci Ther. 2018; 24:1286–98. 10.1111/cns.13058 30596401 PMC6490010

[24] Lipinski CA, Lombardo F, Dominy BW, Feeney PJ. Experimental and computational approaches to estimate solubility and permeability in drug discovery and development settings. Adv Drug Deliv Rev. 2001; 46:3–26. 10.1016/s0169-409x(00)00129-0 11259830

[25] Hitchcock SA, Pennington LD. Structure-brain exposure relationships. J Med Chem. 2006; 49:7559–83. 10.1021/jm060642i 17181137

[26] Liu H, Wang L, Lv M, Pei R, Li P, Pei Z, Wang Y, Su W, Xie XQ. AlzPlatform: an Alzheimer's disease domain-specific chemogenomics knowledgebase for polypharmacology and target identification research. J Chem Inf Model. 2014; 54:1050–60. 10.1021/ci500004h 24597646 PMC4010297

[27] Yang F, Lim GP, Begum AN, Ubeda OJ, Simmons MR, Ambegaokar SS, Chen PP, Kayed R, Glabe CG, Frautschy SA, Cole GM. Curcumin inhibits formation of amyloid beta oligomers and fibrils, binds plaques, and reduces amyloid in vivo. J Biol Chem. 2005; 280:5892–901. 10.1074/jbc.M404751200 15590663

[28] Zuk M, Kulma A, Dymińska L, Szołtysek K, Prescha A, Hanuza J, Szopa J. Flavonoid engineering of flax potentiate its biotechnological application. BMC Biotechnol. 2011; 11:10. 10.1186/1472-6750-11-10 21276227 PMC3040132

[29] Chang KH, Chiu YJ, Chen SL, Huang CH, Lin CH, Lin TH, Lee CM, Ramesh C, Wu CH, Huang CC, Fung HC, Chen YC, Lin JY, et al. The potential of synthetic indolylquinoline derivatives for Aβ aggregation reduction by chemical chaperone activity. Neuropharmacology. 2016; 101:309–19. 10.1016/j.neuropharm.2015.09.005 26362358

[30] Zhao T, Zeng Y, Kermode AR. A plant cell-based system that predicts aβ42 misfolding: potential as a drug discovery tool for Alzheimer's disease. Mol Genet Metab. 2012; 107:571–9. 10.1016/j.ymgme.2012.08.010 22944366

[31] Thapa A, Jett SD, Chi EY. Curcumin Attenuates Amyloid-β Aggregate Toxicity and Modulates Amyloid-β Aggregation Pathway. ACS Chem Neurosci. 2016; 7:56–68. 10.1021/acschemneuro.5b00214 26529184

[32] Di L, Kerns EH, Fan K, McConnell OJ, Carter GT. High throughput artificial membrane permeability assay for blood-brain barrier. Eur J Med Chem. 2003; 38:223–32. 10.1016/s0223-5234(03)00012-6 12667689

[33] Di L, Kerns EH, Bezar IF, Petusky SL, Huang Y. Comparison of blood-brain barrier permeability assays: in situ brain perfusion, MDR1-MDCKII and PAMPA-BBB. J Pharm Sci. 2009; 98:1980–91. 10.1002/jps.21580 18837012

[34] Lin TH, Chiu YJ, Lin CH, Lin CY, Chao CY, Chen YC, Yang SM, Lin W, Mei Hsieh-Li H, Wu YR, Chang KH, Lee-Chen GJ, Chen CM. Exploration of multi-target effects of 3-benzoyl-5-hydroxychromen-2-one in Alzheimer's disease cell and mouse models. Aging Cell. 2020; 19:e13169. 10.1111/acel.13169 32496635 PMC7433010

[35] Nagahara AH, Mateling M, Kovacs I, Wang L, Eggert S, Rockenstein E, Koo EH, Masliah E, Tuszynski MH. Early BDNF treatment ameliorates cell loss in the entorhinal cortex of APP transgenic mice. J Neurosci. 2013; 33:15596–602. 10.1523/JNEUROSCI.5195-12.2013 24068826 PMC3782628

[36] Jiao SS, Shen LL, Zhu C, Bu XL, Liu YH, Liu CH, Yao XQ, Zhang LL, Zhou HD, Walker DG, Tan J, Götz J, Zhou XF, Wang YJ. Brain-derived neurotrophic factor protects against tau-related neurodegeneration of Alzheimer's disease. Transl Psychiatry. 2016; 6:e907. 10.1038/tp.2016.186 27701410 PMC5315549

[37] Eremenko E, Mittal K, Berner O, Kamenetsky N, Nemirovsky A, Elyahu Y, Monsonego A. BDNF-producing, amyloid β-specific CD4 T cells as targeted drug-delivery vehicles in Alzheimer's disease. EBioMedicine. 2019; 43:424–34. 10.1016/j.ebiom.2019.04.019 31085101 PMC6557914

[38] Park H, Poo MM. Neurotrophin regulation of neural circuit development and function. Nat Rev Neurosci. 2013; 14:7–23. 10.1038/nrn3379 23254191

[39] Sala C, Segal M. Dendritic spines: the locus of structural and functional plasticity. Physiol Rev. 2014; 94:141–88. 10.1152/physrev.00012.2013 24382885

[40] Yan Y, Eipper BA, Mains RE. Kalirin is required for BDNF-TrkB stimulated neurite outgrowth and branching. Neuropharmacology. 2016; 107:227–38. 10.1016/j.neuropharm.2016.03.050 27036892 PMC4912856

[41] Wang B, Wu N, Liang F, Zhang S, Ni W, Cao Y, Xia D, Xi H. 7,8-dihydroxyflavone, a small-molecule tropomyosin-related kinase B (TrkB) agonist, attenuates cerebral ischemia and reperfusion injury in rats. J Mol Histol. 2014; 45:129–40. 10.1007/s10735-013-9539-y 24045895

[42] Xhima K, Markham-Coultes K, Nedev H, Heinen S, Saragovi HU, Hynynen K, Aubert I. Focused ultrasound delivery of a selective TrkA agonist rescues cholinergic function in a mouse model of Alzheimer's disease. Sci Adv. 2020; 6:eaax6646. 10.1126/sciadv.aax6646 32010781 PMC6976301

[43] Gonzalez S, McHugh TLM, Yang T, Syriani W, Massa SM, Longo FM, Simmons DA. Small molecule modulation of TrkB and TrkC neurotrophin receptors prevents cholinergic neuron atrophy in an Alzheimer's disease mouse model at an advanced pathological stage. Neurobiol Dis. 2022; 162:105563. 10.1016/j.nbd.2021.105563 34838668

[44] Numakawa T, Suzuki S, Kumamaru E, Adachi N, Richards M, Kunugi H. BDNF function and intracellular signaling in neurons. Histol Histopathol. 2010; 25:237–58. 10.14670/HH-25.237 20017110

[45] Cheng CY, Lin JG, Tang NY, Kao ST, Hsieh CL. Electroacupuncture at different frequencies (5Hz and 25Hz) ameliorates cerebral ischemia-reperfusion injury in rats: possible involvement of p38 MAPK-mediated anti-apoptotic signaling pathways. BMC Complement Altern Med. 2015; 15:241. 10.1186/s12906-015-0752-y 26187498 PMC4506591

[46] Huang CY, Liou YF, Chung SY, Lin WY, Jong GP, Kuo CH, Tsai FJ, Cheng YC, Cheng FC, Lin JY. Role of ERK signaling in the neuroprotective efficacy of magnesium sulfate treatment during focal cerebral ischemia in the gerbil cortex. Chin J Physiol. 2010; 53:299–309. 10.4077/cjp.2010.amk063 21793341

[47] Wang JM, Yang LH, Zhang YY, Niu CL, Cui Y, Feng WS, Wang GF. BDNF and COX-2 participate in anti-depressive mechanisms of catalpol in rats undergoing chronic unpredictable mild stress. Physiol Behav. 2015; 151:360–8. 10.1016/j.physbeh.2015.08.008 26255123

[48] Pillai A, Veeranan-Karmegam R, Dhandapani KM, Mahadik SP. Cystamine prevents haloperidol-induced decrease of BDNF/TrkB signaling in mouse frontal cortex. J Neurochem. 2008; 107:941–51. 10.1111/j.1471-4159.2008.05665.x 18786174

[49] Fonteles AA, de Souza CM, de Sousa Neves JC, Menezes AP, Santos do Carmo MR, Fernandes FD, de Araújo PR, de Andrade GM. Rosmarinic acid prevents against memory deficits in ischemic mice. Behav Brain Res. 2016; 297:91–103. 10.1016/j.bbr.2015.09.029 26456521

[50] Lee B, Sur B, Cho SG, Yeom M, Shim I, Lee H, Hahm DH. Wogonin Attenuates Hippocampal Neuronal Loss and Cognitive Dysfunction in Trimethyltin-Intoxicated Rats. Biomol Ther (Seoul). 2016; 24:328–37. 10.4062/biomolther.2015.152 27133262 PMC4859797

[51] Zhang Z, Liu X, Schroeder JP, Chan CB, Song M, Yu SP, Weinshenker D, Ye K. 7,8-dihydroxyflavone prevents synaptic loss and memory deficits in a mouse model of Alzheimer's disease. Neuropsychopharmacology. 2014; 39:638–50. 10.1038/npp.2013.243 24022672 PMC3895241

[52] Gao L, Tian M, Zhao HY, Xu QQ, Huang YM, Si QC, Tian Q, Wu QM, Hu XM, Sun LB, McClintock SM, Zeng Y. TrkB activation by 7, 8-dihydroxyflavone increases synapse AMPA subunits and ameliorates spatial memory deficits in a mouse model of Alzheimer's disease. J Neurochem. 2016; 136:620–36. 10.1111/jnc.13432 26577931

[53] Chen J, Chua KW, Chua CC, Yu H, Pei A, Chua BH, Hamdy RC, Xu X, Liu CF. Antioxidant activity of 7,8-dihydroxyflavone provides neuroprotection against glutamate-induced toxicity. Neurosci Lett. 2011; 499:181–5. 10.1016/j.neulet.2011.05.054 21651962

[54] Han X, Zhu S, Wang B, Chen L, Li R, Yao W, Qu Z. Antioxidant action of 7,8-dihydroxyflavone protects PC12 cells against 6-hydroxydopamine-induced cytotoxicity. Neurochem Int. 2014; 64:18–23. 10.1016/j.neuint.2013.10.018 24220540

[55] Li XH, Dai CF, Chen L, Zhou WT, Han HL, Dong ZF. 7,8-dihydroxyflavone Ameliorates Motor Deficits Via Suppressing α-synuclein Expression and Oxidative Stress in the MPTP-induced Mouse Model of Parkinson's Disease. CNS Neurosci Ther. 2016; 22:617–24. 10.1111/cns.12555 27079181 PMC6492848

[56] Liu X, Obianyo O, Chan CB, Huang J, Xue S, Yang JJ, Zeng F, Goodman M, Ye K. Biochemical and biophysical investigation of the brain-derived neurotrophic factor mimetic 7,8-dihydroxyflavone in the binding and activation of the TrkB receptor. J Biol Chem. 2014; 289:27571–84. 10.1074/jbc.M114.562561 25143381 PMC4183797

[57] Liu C, Chan CB, Ye K. 7,8-dihydroxyflavone, a small molecular TrkB agonist, is useful for treating various BDNF-implicated human disorders. Transl Neurodegener. 2016; 5:2. 10.1186/s40035-015-0048-7 26740873 PMC4702337

[58] Massa SM, Yang T, Xie Y, Shi J, Bilgen M, Joyce JN, Nehama D, Rajadas J, Longo FM. Small molecule BDNF mimetics activate TrkB signaling and prevent neuronal degeneration in rodents. J Clin Invest. 2010; 120:1774–85. 10.1172/JCI41356 20407211 PMC2860903

[59] Biancalana M, Koide S. Molecular mechanism of Thioflavin-T binding to amyloid fibrils. Biochim Biophys Acta. 2010; 1804:1405–12. 10.1016/j.bbapap.2010.04.001 20399286 PMC2880406

[60] Chiu YJ, Lin TH, Chen CM, Lin CH, Teng YS, Lin CY, Sun YC, Hsieh-Li HM, Su MT, Lee-Chen GJ, Lin W, Chang KH. Novel Synthetic Coumarin-Chalcone Derivative (E)-3-(3-(4-(Dimethylamino)Phenyl)Acryloyl)-4-Hydroxy-2*H*-Chromen-2-One Activates CREB-Mediated Neuroprotection in Aβ and Tau Cell Models of Alzheimer's Disease. Oxid Med Cell Longev. 2021; 2021:3058861. 10.1155/2021/3058861 34812274 PMC8605905

[61] Li N, Liu JH, Zhang J, Yu BY. Comparative evaluation of cytotoxicity and antioxidative activity of 20 flavonoids. J Agric Food Chem. 2008; 56:3876–83. 10.1021/jf073520n 18433100

[62] Verdonk ML, Cole JC, Hartshorn MJ, Murray CW, Taylor RD. Improved protein-ligand docking using GOLD. Proteins. 2003; 52:609–23. 10.1002/prot.10465 12910460

[63] Nissink JW, Murray C, Hartshorn M, Verdonk ML, Cole JC, Taylor R. A new test set for validating predictions of protein-ligand interaction. Proteins. 2002; 49:457–71. 10.1002/prot.10232 12402356

[64] Lin CH, Hsieh YS, Wu YR, Hsu CJ, Chen HC, Huang WH, Chang KH, Hsieh-Li HM, Su MT, Sun YC, Lee GC, Lee-Chen GJ. Identifying GSK-3β kinase inhibitors of Alzheimer's disease: Virtual screening, enzyme, and cell assays. Eur J Pharm Sci. 2016; 89:11–9. 10.1016/j.ejps.2016.04.012 27094783

[65] Banfield MJ, Naylor RL, Robertson AG, Allen SJ, Dawbarn D, Brady RL. Specificity in Trk receptor:neurotrophin interactions: the crystal structure of TrkB-d5 in complex with neurotrophin-4/5. Structure. 2001; 9:1191–9. 10.1016/s0969-2126(01)00681-5 11738045

[66] Urfer R, Tsoulfas P, O'Connell L, Shelton DL, Parada LF, Presta LG. An immunoglobulin-like domain determines the specificity of neurotrophin receptors. EMBO J. 1995; 14:2795–805. 10.1002/j.1460-2075.1995.tb07279.x 7796806 PMC398398

[67] Chitranshi N, Gupta V, Kumar S, Graham SL. Exploring the Molecular Interactions of 7,8-Dihydroxyflavone and Its Derivatives with TrkB and VEGFR2 Proteins. Int J Mol Sci. 2015; 16:21087–108. 10.3390/ijms160921087 26404256 PMC4613243

[68] Påhlman S, Ruusala AI, Abrahamsson L, Mattsson ME, Esscher T. Retinoic acid-induced differentiation of cultured human neuroblastoma cells: a comparison with phorbolester-induced differentiation. Cell Differ. 1984; 14:135–44. 10.1016/0045-6039(84)90038-1 6467378

[69] Huang CC, Chang KH, Chiu YJ, Chen YR, Lung TH, Hsieh-Li HM, Su MT, Sun YC, Chen CM, Lin W, Lee-Chen GJ. Multi-Target Effects of Novel Synthetic Coumarin Derivatives Protecting Aβ-GFP SH-SY5Y Cells against Aβ Toxicity. Cells. 2021; 10:3095. 10.3390/cells10113095 34831318 PMC8619673

[70] Ottaviani G, Martel S, Escarala C, Nicolle E, Carrupt PA. The PAMPA technique as a HTS tool for partition coefficients determination in different solvent/water systems. Eur J Pharm Sci. 2008; 35:68–75. 10.1016/j.ejps.2008.06.006 18620049

